# Effectsof growth‐promoting rhizobacteria on maize growth and rhizosphere microbial community under conservation tillage in Northeast China

**DOI:** 10.1111/1751-7915.13693

**Published:** 2020-11-09

**Authors:** La Chen, Zhanhong Hao, Keke Li, Ye Sha, Entao Wang, Xinhua Sui, Guohua Mi, Changfu Tian, Wenxin Chen

**Affiliations:** ^1^ State Key Laboratory for Agrobiotechnology Key Laboratory of Soil Microbiology, Ministry of Agriculture College of Biological Sciences China Agricultural University Beijing 100193 China; ^2^ Key Laboratory of Plant‐Soil Interactions Ministry of Education College of Resources and Environmental Sciences China Agricultural University Beijing 100193 China; ^3^ Escuela Nacional de Ciencias Biologicas Instituto Politecnico Nacional Mexico D.F. 11340 Mexico

## Abstract

Conservation tillage in conjunction with straw mulching is a sustainable agricultural approach. However, straw mulching reduces the soil temperature, inhibits early maize growth and reduces grain yield in cold regions. To address this problem, we investigated the effects of inoculation of plant growth‐promoting rhizobacteria (PGPR) on maize growth and rhizosphere microbial communities under conservation tillage in Northeast China. The PGPR strains *Sinorhizobium* sp. A15, *Bacillus* sp. A28, *Sphingomonas* sp. A55 and *Enterobacter* sp. P24 were isolated from the maize rhizosphere in the same area and inoculated separately. Inoculation of these strains significantly enhanced maize growth, and the strains A15, A28 and A55 significantly increased grain yield by as much as 22%–29%. Real‐time quantitative PCR and high‐throughput sequencing showed that separate inoculation with the four strains increased the abundance and species richness of bacteria in the maize rhizosphere. Notably, the relative abundance of *Acidobacteria*_Subgroup_6, *Chloroflexi*_KD4‐96, and *Verrucomicrobiae* at the class level and *Mucilaginibacter* at the genus level were positively correlated with maize biomass and yield. Inoculation with PGPR shows potential for improvement of maize production under conservation tillage in cold regions by regulating the rhizosphere bacterial community structure and by direct stimulation of plant growth.

## Graphical abstract

The PGPR strains *Sinorhizobium* sp. A15, *Bacillus* sp. A28, *Sphingomonas* sp. A55, and *Enterobacter* sp. P24 were isolated from the maize rhizosphere in Northeast China and inoculated separately; Inoculation of these strains significantly enhanced maize growth, and the strains A15, A28, and A55 significantly increased grain yield by as much as 22%–29%; Inoculation of these strains increased the abundance and species richness of bacteria in the maize rhizosphere, and the relative abundance of *Acidobacteria*_Subgroup_6, *Chloroflexi*_KD4‐96, and *Verrucomicrobiae* at the class level and *Mucilaginibacter* at the genus level were positively correlated with maize biomass and yield.

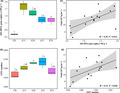

## Introduction

Maize, a major staple food crop grown worldwide, accounted for 38% of the world's cereal production in 2017 (FAOSTAT, 2017) and is of vital importance in ensuring global food security. Northeast China is considered to be among the three 'golden corn belts' in the world and accounts for approximately 40% of the national maize production in China (NBSC, 2019). The traditional cultivation practice in this area is ridge‐till, which creates favourable soil conditions for maize growth by implementing a sequence of deep soil tilling, rotary tillage and ridging. However, with global climate change, rainfall in the maize growing season in this region has generally decreased over the last five decades and drought has increased in frequency, especially in spring (Zhang, 2004; Song *et al*., 2014; Yu *et al*., 2014). The frequent windy weather in winter and spring, and excessive soil disturbance caused by repeated tillage, has resulted in severe wind erosion of the topsoil (Zhang *et al*., 2015; Yan *et al*., 2018). In addition, water erosion of soil on slopes in this region accompanies summer rainstorms (An *et al*., 2014). All these factors contribute to a decline in soil fertility (Liu *et al*., 2010), similar to the situation observed in the major maize‐producing areas in the United States and Canada (Hermawan and Bomke, 1997; Montgomery, 2007; Yoo and Wander, 2008).

Conservation tillage (e.g. no‐till and strip‐till) in conjunction with straw mulching has been widely applied in the maize‐producing areas of the United States and Canada (Schlesinger, 1999; Awada *et al*., 2014). This approach is effective in protecting soil from wind and water erosion, and enhances the drought resistance of soil (Doran *et al*., 1998; Six *et al*., 2004; Dumanski and Peiretti, 2013). However, in cold‐climate areas, straw mulching leads to a lower soil temperature and in turn depresses the growth of maize seedlings (Gupta *et al*., 1988; Moroizumi and Horino, 2002). Strip tillage with straw mulching, a practice developed as a form of no‐tillage, can improve maize growth through raising the soil temperature of the planting belt (Vyn and Raimbault, 1992; Morrison, 2002; Licht and Al‐Kaisi, 2005). Nevertheless, it is usually difficult to attain the yields of the traditional tillage system, so conservation tillage results in reduced maize production in many cases (West *et al*., 1996; Chen *et al*., 2011; Shen *et al*., 2018), which hinders widespread application of this cultivation model. Therefore, promoting growth and increasing the yield of maize crops are the primary obstacles for popularization and wide application of the strip‐till practice in cold‐climate areas.

Plant growth‐promoting rhizobacteria (PGPR) can promote plant growth by providing nutrients, synthesizing phytohormones, defending against pathogens, reducing stress, alleviating soil contamination with heavy metals (Kloepper and Schroth, 1978; Bardi and Malusá, 2012; Cawoy *et al*., 2015; Wang *et al*., 2015) or improving the microbial community structure of the rhizosphere (Vessey, 2003; Malusa and Vassilev, 2014). For example, Wang *et al*. (2018) reported that inoculation with a mixture of *Ensifer* sp. NYM3, *Acinetobacter* sp. P16 and *Flavobacterium* sp. KYM3 increased cucumber yield and significantly affected the indigenous soil bacterial community; notably, the relative abundance of *Gammaproteobacteria*, *Acidobacteria*, *Nitrospirae* and *Armatimonadetes* increased significantly. Zhang *et al*. (2019b) observed that a consortium of PGPR for inoculation of sweet pepper suppresses disease incidence by altering the rhizosphere microbiota, such as increasing the relative abundance of *Burkholderia*, *Comamonas* and *Ramlibacter*.

In the field, the ability of PGPR to promote growth is affected by soil characteristics (Egamberdiyeva, 2007; Sessitsch *et al*., 2019). Given that tillage practice can change soil characteristics (Alvarez and Steinbach, 2009), it may be expected that the effects of PGPR on plants and the soil bacterial community are affected by conservation tillage (Mbuthia *et al*., 2015). Florine *et al*. (2017) observed that in the soils of *Vicia faba* and *Triticum aestivum* fields, conventional tillage enriched copiotrophic bacteria, whereas reduced tillage enriched oligotrophic bacteria. Wang *et al*. (2020b) reported that the assembly and composition of bacterial communities in the rhizosphere soil of wheat under different tillage practices differed significantly, and the rhizosphere bacterial communities were more stable under no‐tillage than those under plough tillage. Similar results were observed in the rhizosphere of maize (Wang *et al*., 2020a). Therefore, it is important to study the growth‐promotive effect of PGPR inoculation under different tillage practices. However, few studies have investigated the effect of PGPR on the promotion of maize growth under conservation tillage, especially the influence of PGPR inoculation on the microbial community structure of the maize rhizosphere.

In our previous study, we isolated PGPR from the maize rhizosphere in Northeast China. Four PGPR strains (A15, A55, A28 and P24) showed plant growth‐promoting properties, and their inoculation positively influenced maize growth and grain yield (unpubl. data in Tables S1–S3). In the present study, we tested the effects of these PGPR strains in promoting maize growth and regulating microbial communities in the maize rhizosphere under a conservation tillage (strip tillage) system. The aim was to evaluate the potential of PGPR inoculation for improvement of maize production under conservation tillage in a cold region.

## Results

### Bacterial isolation from the rhizosphere and PGPR selection

In total, 131 purified colonies were isolated from maize rhizosphere soil collected from the experimental field in this study. Based upon the laboratory screening, 14 isolates showed plant growth‐promoting properties, such as indole‐3‐acetic acid (IAA) synthesis, phosphate solubilization, potassium (K) solubilization and siderophore release (Table S1). These 14 strains were further screened in the same area in a two‐year field inoculation experiment on maize grown under a conventional ridge‐till system with reduced nitrogen (N; 50%) and zero phosphorus (P) fertilizer application. Inoculation of the four PGPR strains (A15, A28, A55 and P24) significantly promoted maize growth at different developmental stages (Table S2). The grain yield of inoculated maize under reduced N and/or zero P was significantly greater than that of the reduced fertilization control (CK1) (Table S3) and was similar to that under high (conventional) N and P fertilization (CK2). Therefore, these four PGPR strains were used in the current experiment to evaluate their effects on maize grown under a conservation tillage system. Based upon 16S rRNA gene sequence analysis, the four selected PGPR isolates were identified as *Sinorhizobium* sp. A15 (MT956581), *Bacillus* sp. A28 (MN905525), *Sphingomonas* sp. A55 (MN905523) and *Enterobacter* sp. P24 (MN905526) respectively (Fig. S1). The four strains all synthesized IAA, A55 and A28 were siderophore producers, A28 solubilized K, and P24 solubilized inorganic and organic phosphate (Table S1).

### Plant growth traits and grain yield

The growth status of maize plants at the jointing stage is summarized in Table 1. Compared with the non‐inoculation control, separate inoculation of the four PGPR strains significantly (*P* < 0.05) promoted plant height (by 41.6%–47.2%), shoot dry weight (by 70.9%–86.6%), root dry weight (by 61.7%–75.6%) and leaf area index (by 72.9%–82.4%). Notably, grain yield increased in response to inoculation of the four strains; in particular, inoculation of A15, A55 and A28 significantly (*P* < 0.05) increased yield by 22.2%–28.9% compared with the non‐inoculation control (Table 1). The increase in yield was associated with number of ears, number of grains per ear and hundred‐grain dry weight collectively, which increased by 1.9%–6.1%, 0.9%–2.3% and 0.8%–3.5%, respectively, but no differences attained significance (Table 1). These results indicated that the increase in grain yield in response to PGPR inoculation was due to improvement in overall maize growth.

**Table 1:** Effect of inoculation of plant growth‐promoting rhizobacteria on maize growth traits at the jointing stage and grain yield at the maturation stage.

Treatments	Growth features (per plant) at jointing stage				Grain yield at maturation stage			
	Plant height (cm)	Shoot dry weight (g)	Root dry weight (g)	Leaf area index	Ear numbers (10^4^ ha^‐1^)	Hundred‐grain dry weight (g)	Grain number per ear	Yield (10^3^ kg ha^−1^)
A15	131 ± 2a	23.7 ± 1.2a	3.16 ± 0.12a	1.55 ± 0.04a	6.83 ± 0.10a	28.3 ± 1.2a	533 ± 11a	11.6 ± 1.1a
A55	128 ± 2a	22.1 ± 1.1a	3.08 ± 0.13a	1.49 ± 0.04a	6.56 ± 0.15a	28.1 ± 1.1a	540 ± 0a	11.4 ± 0.3a
A28	126 ± 3a	22.0 ± 1.2a	2.97 ± 0.11a	1.48 ± 0.03a	6.72 ± 0.11a	27.6 ± 0.4a	537 ± 11a	11.0 ± 0.5a
P24	126 ± 2a	21.7 ± 1.1a	2.91 ± 0.10a	1.47 ± 0.05a	6.22 ± 0.34a	28.4 ± 0.7a	541 ± 9a	10.8 ± 0.2ab
CK	89 ± 2b	12.7 ± 0.8b	1.80 ± 0.11b	0.85 ± 0.04b	6.44 ± 0.31a	27.4 ± 0.4a	529 ± 16a	9.0 ± 0.5b

Data are the average ± standard error (*n* = 4); CK: non‐inoculation control; A15, A28, A55 and P24 present the inoculation treatments with strains A15, A28, A55 and P24 respectively; different letters in the same column indicate a significant difference among treatments (*P* = 0.05).

### Rhizobacterial abundance/species richness and their correlations with grain yield

**Fig. 1:**
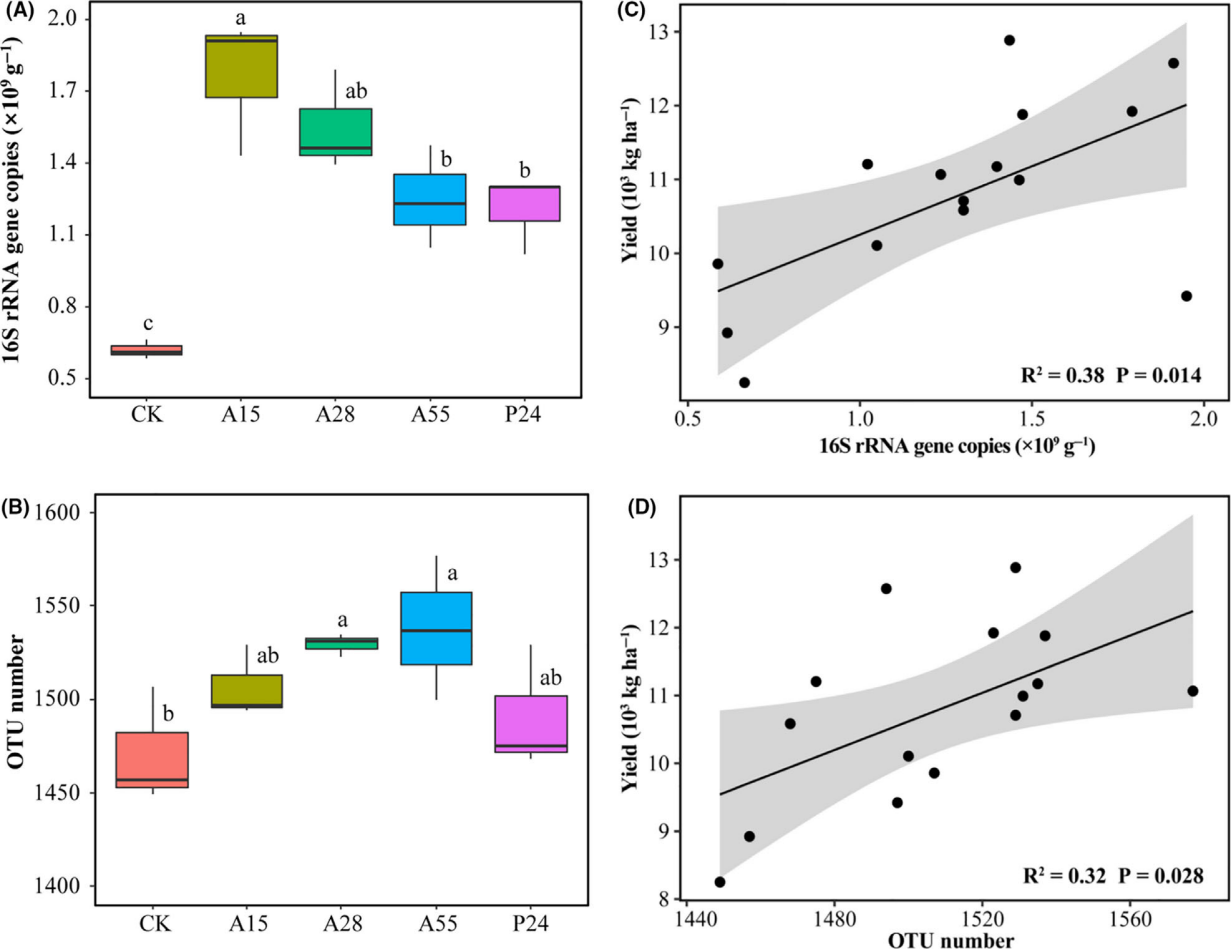
Rhizobacterial abundance/species richness and their correlations with grain yield. Copy numbers of the 16S rRNA gene (A) and number of operational taxonomic units (OTUs) (B) among all treatments; relationship of 16S rRNA gene copy numbers (C) and number of OTUs (D) with grain yield; grey shading represents the 95% confidence interval; CK: non‐inoculation control; A15, A28, A55 and P24 present the inoculation treatments with strains A15, A28, A55 and P24 respectively; different letters above each box indicate a significant difference among treatments (*P* = 0.05).

The number of copies of the 16S rRNA gene in the rhizosphere microbial communities was 0.62–1.77 × 10^9^ copies g^−1^ soil (Fig. 1a). Compared with the non‐inoculation control, the four PGPR inoculation treatments significantly increased the copy numbers of the 16S rRNA gene (*P* < 0.05). Inoculation of A15 resulted in the highest copy numbers of the 16S rRNA gene, which was 2.85 times that in the non‐inoculation control.

A total of 817,217 high‐quality sequences were obtained from 20 soil DNA samples with 20 997–53 812 sequences per sample (Table S4). For each sample, 20 000 sequences were randomly rarified, and the number of operational taxonomic units (OTUs) per sample ranged between 1403 and 1577 according to a 97% similarity threshold for species (Table S4). Rarefaction curves (Fig. S2), which showed the number of OTUs and diversity index as a function of sequencing effort, indicated that the sequencing depth was sufficient to reveal the bacterial diversity in the samples. In addition, the coverage of each sample was more than 98%, which indicated that the obtained sequence objectively and accurately reflected the richness and diversity of the bacterial communities. Compared with the non‐inoculation control, the PGPR inoculation treatments increased the species richness of bacteria (number of OTUs), and the response to inoculation with A28 and A55 attained significance (*P* < 0.05) (Fig. 1B).

Correlation analysis showed that the copy number of the 16S rRNA gene and the OTU number of the bacterial communities were positively correlated with grain yield at maturation of maize (*P* < 0.05) (Fig. 1C and D). These results demonstrated that the increase in grain yield stimulated by PGPR inoculation might be associated with the increase in abundance and species richness of rhizobacteria.

### Structure and composition of bacterial communities

**Fig. 2:**
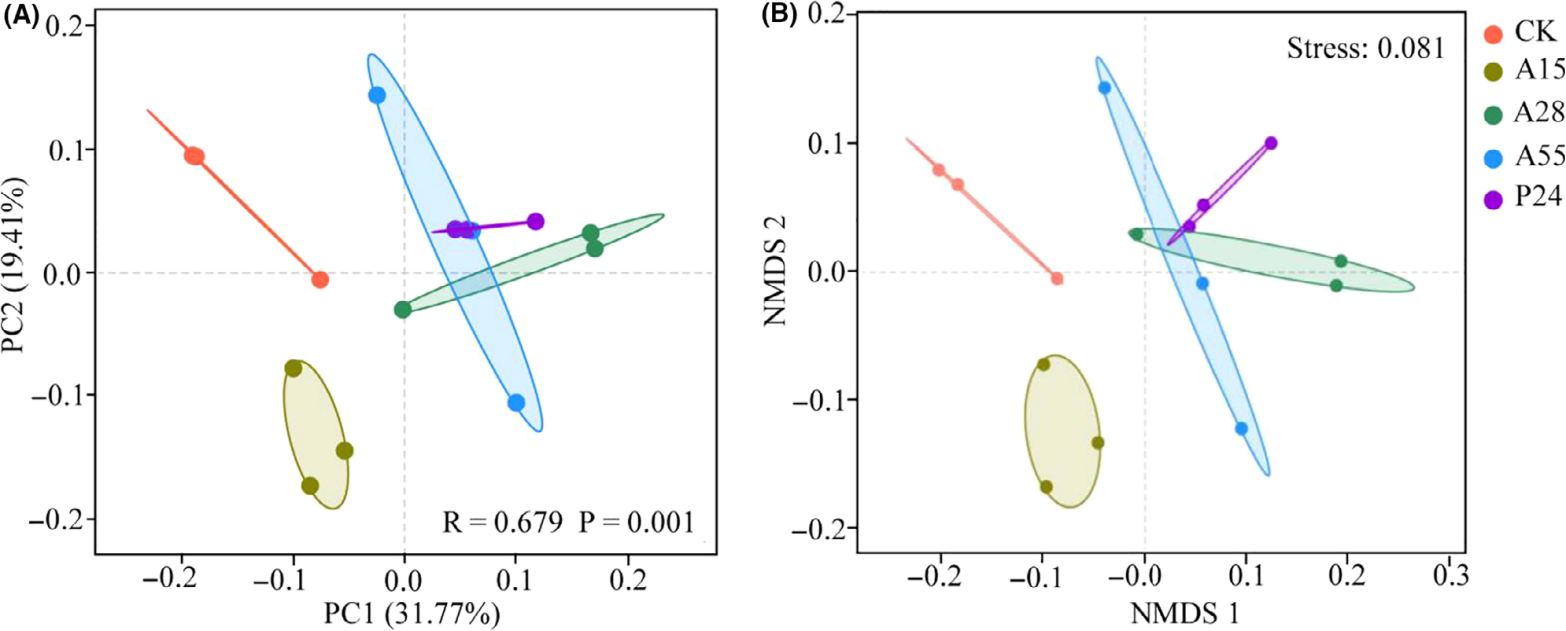
Principal coordinate analysis (PCoA) (A) and non‐metric multidimensional scaling (NMDS) (B) analysis of bacterial community composition based on Bray–Curtis distances. The statistical significance of differences in all treatments was assessed by analysis of similarity (Anosim) with *R* = 0.679, *P* = 0.001; CK: non‐inoculation control; A15, A28, A55 and P24 present the inoculation treatments with strains A15, A28, A55 and P24 respectively.

To visualize differences in the structure of bacterial communities among all treatments, principal coordinate analysis (PCoA), similarity analysis (Anosim) and non‐metric multidimensional scaling (NMDS) were conducted based on Bray–Curtis distances. The PCoA plots showed distinct clustering of the rhizosphere bacterial communities in accordance with the PGPR strain treatments; the first principal axis (31.77%) and second principal axis (19.41%) explained 51.18% of the total variation among the bacterial communities (Fig. 2A). A similar result was observed in the NMDS plots (stress = 0.081) (Fig. 2B). Anosim analysis indicated significant difference in the bacterial community structure among the all treatments (*R* = 0.679, *P* < 0.05) (Table S5). The bacterial community structure in each inoculation treatment except of A55 was significantly different from that in its non‐inoculation control (*P* < 0.05). The bacterial community structure in A15 treatment was significantly different from that in A55 and P24 treatment (*P* < 0.05) (Table S5).

**Fig. 3:**
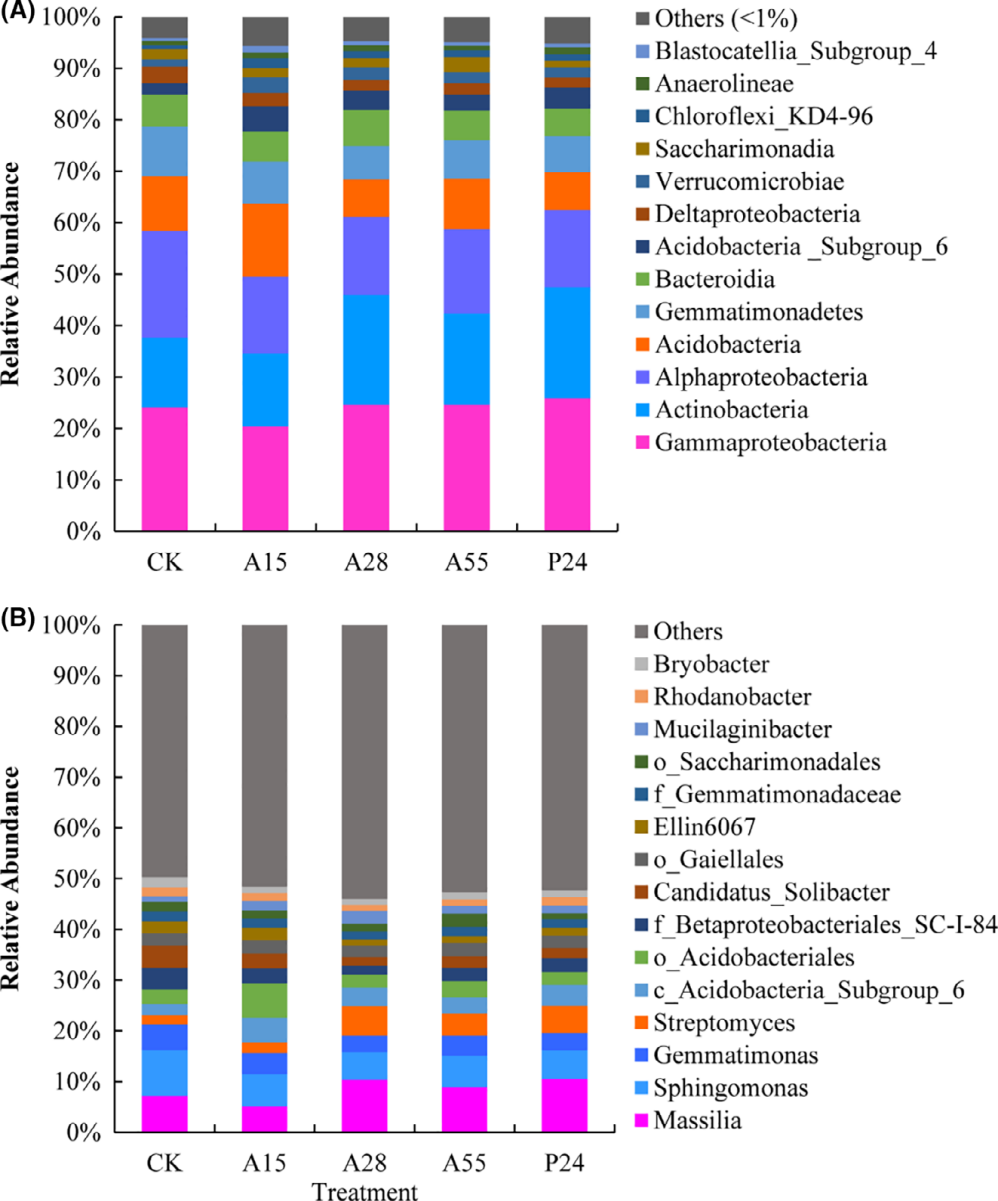
Relative abundance of predominant bacteria at class level (relative abundance > 1%) (A) and genus level (the 15 most abundant) (B) for each treatment. CK: non‐inoculation control; A15, A28, A55 and P24 present the inoculation treatments with strains A15, A28, A55 and P24 respectively.

The 16S rRNA gene sequences of the test samples were classified into 21 phyla, 48 classes, 113 orders, 200 families and 359 genera. The 13 predominant bacterial classes (relative abundance > 1%) are shown in Figure 3A, which comprised *Gammaproteobacteria*, *Actinobacteria*, *Alphaproteobacteria*, *Acidobacteria*, *Gemmatimonadetes*, *Bacteroidia*, *Acidobacteria*_Subgroup_6, *Deltaproteobacteria*, *Verrucomicrobiae*, *Saccharimonadia*, *Chloroflexi*_KD4‐96, *Anaerolineae* and *Blastocatellia*_Subgroup_4, and accounted for 94.31%–95.89% of all sequences. In addition, further analysis of the microbial communities was conducted at the genus level. The 15 most abundant bacterial genera are shown in Figure 3B. *Massilia* was the most abundant across all samples, with a relative abundance of 5.11%–10.46%, followed by *Sphingomonas* (5.45%–9.00%) and *Gemmatimona*s (3.33%–5.03%).

The changes in relative abundance of the predominant bacteria at class (Table S6) and genus (Table S7) levels were further analysed using one‐way analysis of variance (ANOVA). Inoculation with the PGPR strains increased the relative abundances of six classes and three genera, comprising the classes *Actinobacteria* (by 4.73%–60.04%), *Acidobacteria*_Subgroup_6 (by 43.97%–121.95%), *Verrucomicrobiae* (by 53.71%–122.40%), *Chloroflexi*_KD4‐96 (by 80.00%–180.47%), *Anaerolineae* (by 11.59%–63.56%), and *Blastocatellia*_Subgroup_4 (by 30.66%–104.01%), and the genera *Streptomyces* (by 8.67%–201.04%), unclassified_c_*Acidobacteria*_Subgroup_6 (by 44.79%–122.967%) and *Mucilaginibacter* (by 33.33%–113.77%). Conversely, the relative abundances of three classes and seven genera were decreased, consisting of the classes *Alphaproteobacteria* (by 20.58%–27.94%), *Gemmatimonadetes* (by 15.04%–33.52%), and *Deltaproteobacteria* (by 18.12%–40.45%), and the genera *Sphingomonas* (by 29.25%–39.40%), *Gemmatimonas* (by 17.32%–32.89%), unclassified_f*_Betaproteobacteriales_*SC‐I‐84 (by 29.88%–56.82%), *Candidatus_Solibacter* (by 36.19%–62.98%), unclassified_f*_Gemmatimonadaceae* (by 3.38%–17.61%), *Rhodanobacter* (by 9.55%–30.40%) and *Bryobacter* (by 28.87%–43.86%). In addition, compared with the non‐inoculation control, the relative abundances of the class *Alphaproteobacteria* and the genera *Sphingomonas*, *Candidatus_Solibacter*, and *Bryobacter* were significantly reduced in response to inoculation with all four PGPR (*P* < 0.05); the class *Deltaproteobacteria* was decreased significantly by A28, A55 and P24 inoculation (*P* < 0.05); relative abundance of the genus *Ellin6067* was decreased significantly by A28 and A55 inoculation (*P* < 0.05); and the class *Gemmatimonadetes* and the genera *Gemmatimonas* and unclassified_f*_Betaproteobacteriales_*SC‐I‐84 were decreased significantly in relative abundance by A28 inoculation (*P* < 0.05) (Tables S6 and S7). By contrast, the relative abundance of the genus *Streptomyces* was significantly increased by A28, A55 and P24 inoculation (*P* < 0.05); the class *Actinobacteria* and the genus *Massilia* were significantly increased in relative abundance by A28 and P24 inoculation (*P* < 0.05); and the class *Chloroflexi*_KD4‐96 and the genus unclassified_o_*Acidobacteriales* were significantly increased in relative abundance by A15 inoculation (*P* < 0.05) (Tables S6 and S7). These results demonstrated that PGPR inoculation may coordinate the ecological functions of rhizosphere microbiota by regulating the relative abundance of the predominant bacteria.

**Fig. 4:**
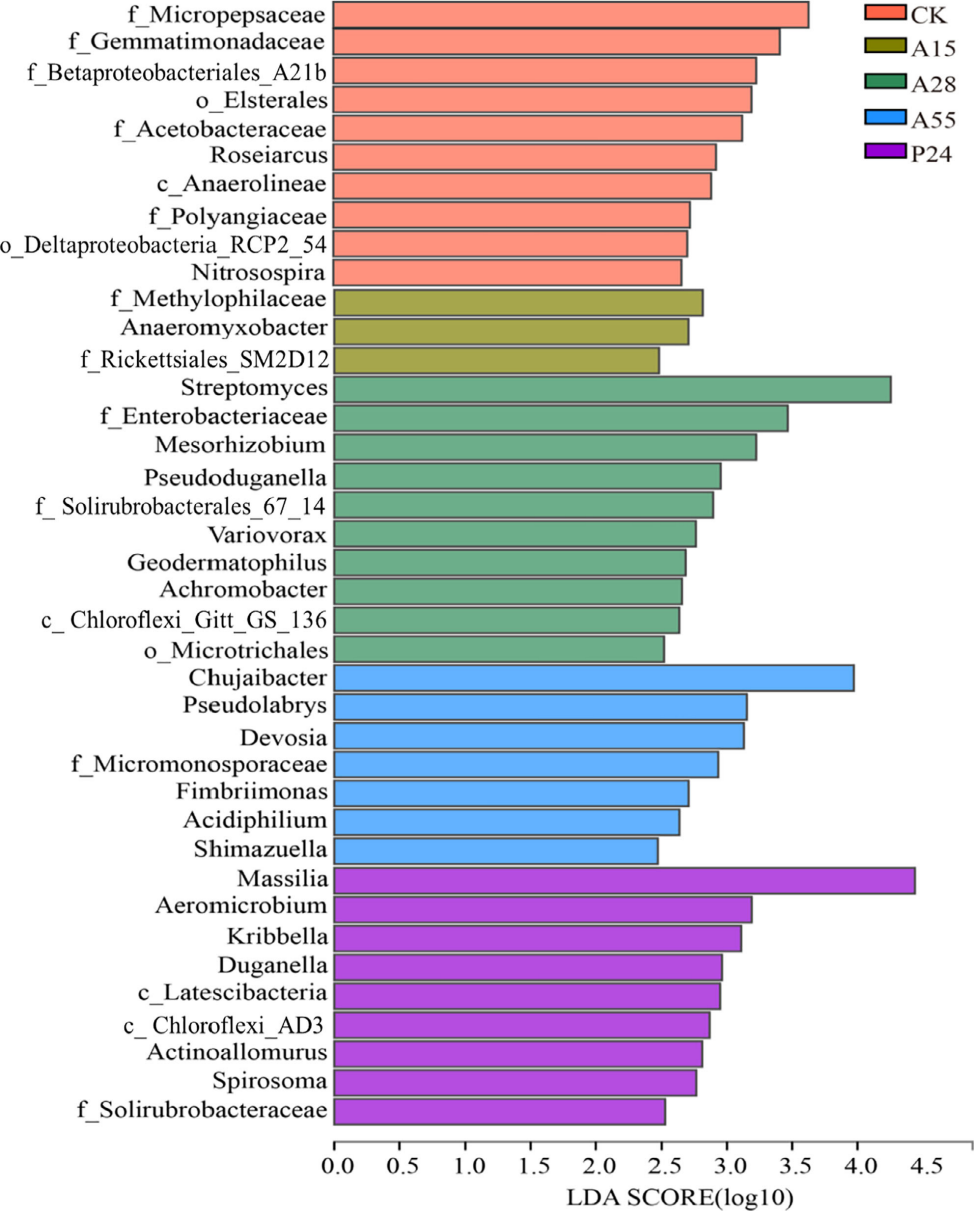
Biomarker bacteria at the genus level for each treatment determined by linear discriminant analysis (LDA) with a threshold value of 2.0. CK: non‐inoculation control; A15, A28, A55 and P24 present the inoculation treatments with strains A15, A28, A55 and P24 respectively.

Linear discriminant analysis effect size (LEfSe) was used to further analyse the biomarker bacteria at genus level among the treatments (Fig. 4, Table S8). Thirty‐nine biomarker bacteria were detected in all treatments, of which the non‐inoculation control and inoculation of A15, A28, A55 and P24 treatments comprised 10, 3, 10, 7 and 9 biomarker bacteria respectively. For example, unclassified_f_*Micropepsaceae* (1.69%), unclassified_f*_Gemmatimonadaceae* (0.74%) and norank_o*_Elsterales* (0.62%) were enriched by the non‐inoculation control; *Anaeromyxobacter* (0.08%), unclassified_f_*Rickettsiales*_SM2D12 (0.05%) and unclassified_f*_Methylophilaceae* (0.04%) were enriched by inoculation with A15; *Streptomyces* (5.79%), unclassified_f*_Enterobacteriaceae* (0.64%) and *Mesorhizobium* (0.64%) were enriched by A28 inoculation; *Chujaibacter* (2.34%), *Pseudolabrys* (0.76%) and *Devosia* (0.59%) were enriched by A55 inoculation; and *Massilia* (10.46%), *Aeromicrobium* (0.54%) and *Kribbella* (0.42%) were enriched by P24 inoculation. The difference in microbial distribution among the treatments reflected the effect of the bacterial inoculants on rhizobacterial populations to some extent.

### Correlation between predominant bacteria and maize growth traits/grain yield

**Fig. 5:**
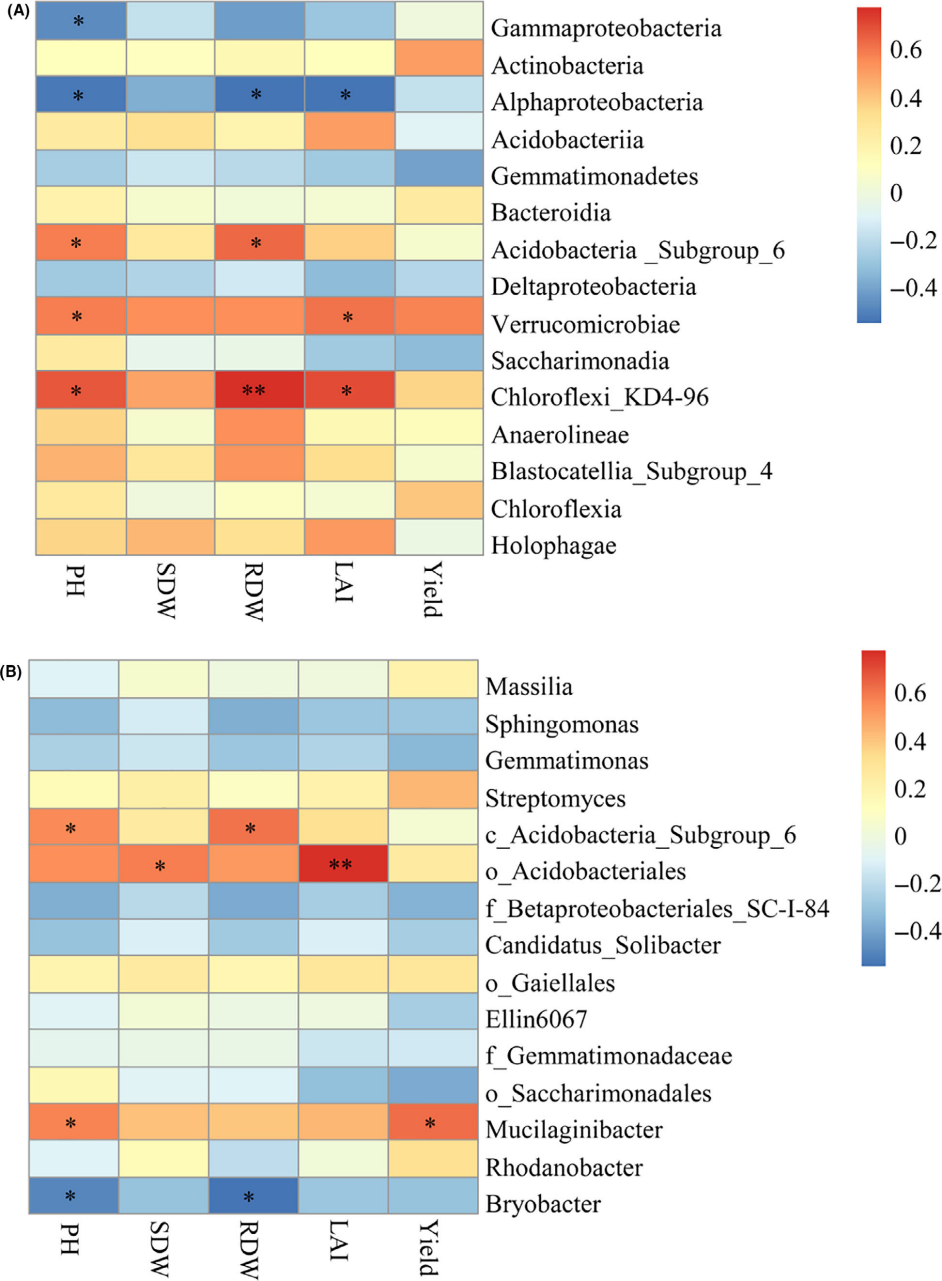
Spearman correlation coefficients between the relative abundance of predominant bacteria at the class level (relative abundance > 1%) (A) and the genus level (the 15 most abundant) (B), and plant height (PH), shoot dry weight (SDW), root dry weight (RDW), leaf area index (LAI) at the jointing stage and grain yield (Yield) at the maturation stage. c_, o_ and f_ were unclassified bacteria at the genus level, representing class, order and family respectively; *0.01 < *P* ≤ 0.05, **P ≤ 0.01.

Spearman correlation coefficients were calculated between the relative abundance of predominant bacteria at class (relative abundance > 1%) and genus (the top 15) levels, and plant height, shoot dry weight, root dry weight, leaf area index at the jointing stage and grain yield at the maturation stage (Fig. 5). At the class level, *Chloroflexi*_KD4‐96 showed a significant positive correlation with plant height, root dry weight and leaf area index (*P* < 0.05)*. Acidobacteria*_Subgroup_6 showed a significant positive correlation with plant height and root dry weight (*P* < 0.05), and *Verrucomicrobiae* was significantly positively correlated with plant height and leaf area index (*P* < 0.05). At the genus level, unclassified_c_*Acidobacteria*_Subgroup_6 and unclassified_o*_Acidobacteriales* showed a significant positive correlation respectively with plant height and root dry weight, and with plant dry weight and leaf area index (*P* < 0.05). *Mucilaginibacter* was significantly positive correlated with plant height and grain yield (*P* < 0.05). The class *Alphaproteobacteria* and the genus *Bryobacter* were negatively correlated with maize plant height and root dry weight (*P* < 0.05).

## Discussion

### Effect of PGPR inoculation on maize growth and yield under conservation tillage

Previous studies have shown that PGPR inoculation may enhance crop yields to different degrees, improve soil fertility, and increase absorption and utilization of soil nutrients (Cassán *et al*., 2009; Díaz‐Zorita and Fernández‐Canigia, 2009; Singh *et al*., 2011). In the warm humid region of Argentina and Brazil, inoculation with different *Azospirillum brasilense* strains isolated from the maize rhizosphere increased maize grain yields by 5%–11% under no‐tillage irrespective of soil type (Salvo *et al*., 2018; Skonieski *et al*., 2019). However, it remains unknown whether PGPR inoculation under conservation tillage is beneficial in cold‐climate areas. In the present research, we studied the growth‐promotive effect of microbial inoculants under strip tillage in black soil in Northeast China (a cold‐climate area). Considering that in a cold region the lower soil temperature caused by conservation tillage is the primary factor limiting growth at the seedling stage, we focused on IAA production in PGPR selection and incorporated l‐tryptophan (a precursor of IAA) in the culture medium for the inoculant, because IAA can stimulate the growth of maize seedlings and in turn enhance the biomass accumulation and grain yield of the treated maize plants (Dubeikovsky *et al*., 1993). The results summarized in Table 1 demonstrated that the PGPR selection strategy was well founded because the growth of maize plants at the jointing stage and the grain yield at the maturation stage were significantly improved by inoculation of the PGPR strains.

Compared with previous results acquired in other climate areas, inoculation with the four PGPR strains in the present study generated a greater increase in grain yield (22%–29%) under strip tillage (Table 1). The enhanced effects might be associated with the fact that the PGPR used in the present study were isolated from the same region, because an inoculant selected from the indigenous microflora might be better adapted and more effective than exogenous bacteria, as reported previously for rhizobial populations (Jia *et al*., 2008). Considering that the present experimental field contained black soil and was located within a 'golden corn belt' that accounts for approximately 40% of the national maize production in China (NBSC, 2019), the present results are important for promotion of PGPR inoculation under conservation tillage in a cold region, although application of the inoculants selected in the present study may be limited to areas with a similar soil type and climate. In addition, many previous studies have revealed that the microbial communities associated with plants (Zhang *et al*., 2011; Román‐Ponce *et al*., 2016) are determined by both the plant species and soil (biotic and abiotic) features. Therefore, a preferable strategy for PGPR selection and application might be to focus on efficient screening of region‐ and plant‐specific PGPR, which may have a stable effect on improvement of crop production. However, by this measure a single PGPR inoculant used in a wide range of environments and for diverse plants would be accompanied by unstable results, as has been observed previously (Martínez‐Viveros *et al*., 2010; Wu *et al*., 2012). The inconsistent results of PGPR inoculation under different experimental conditions have been associated with limited knowledge of the ecology, survival and activity of the inoculant in the rhizosphere (Martínez‐Viveros *et al*., 2010).

### Mechanisms of the PGPR strains to improve maize production

It is common for inoculated bacterial populations to decline rapidly in non‐sterile soils and that re‐inoculation is necessary at intervals during the plant growth period to maintain effective cell densities in the field (Martínez‐Viveros *et al*., 2010). The low abundance of inoculated PGPR strains in the maize rhizosphere detected in the present study was consistent with previous results (Bhattacharyya *et al*., 2018). However, inoculation of the four strains still significantly improved the growth and yield of maize (Table 1), implying that a specific plant growth‐promoting mechanism(s) independent of the inoculant density might function in these strains. In general, two types of mechanisms are involved in the effects of PGPR on plants, namely direct and indirect mechanisms (Martínez‐Viveros *et al*., 2010). Direct mechanisms include production of phytohormones to regulate plant growth, improvement of plant nutritional supply (N fixation and P/K solubilization) and stimulation of systemic disease resistance of plants. Indirect mechanisms refer to biocontrol, such as antagonism to phytopathogens by antibiotic and chitinase production, competition for niches or available iron (by siderophore production) in the rhizosphere.

The four PGPR strains used in this study might involve direct mechanisms because all strains produced IAA and certain strains solubilized phosphate (P24) or K (A28) (Table S1). The strains A55 and A28 showed likely indirect mechanism because they produced siderophores. For effective impacts on plants by solubilization of P and K, or by production of siderophores, the inoculant strains must maintain a reasonable population density of active cells (Martínez‐Viveros *et al*., 2010). However, in the present analysis of the rhizobacteriome by high‐throughput sequencing, the genera that the four inoculated PGPR strains belonged to were not dominant or were not significantly increased by inoculation. Thus, the most plausible mechanism in these four strains for plant growth promotion was the production of IAA.

In addition of the plant growth‐promoting traits of the four inoculated strains, inoculation also apparently regulated both the amount and the community structure of bacteria in the maize rhizosphere (Figs 1, 2, 3, 4), which might be an additional indirect mechanism to improve the growth and yield of maize. Given that rhizosphere bacteria are more closely associated with plant health owing to their unique ecological niche, it is highly meaningful to explore the effect of PGPR inoculation on the rhizosphere microbiome (Berendsen *et al*., 2012; García‐Salamanca *et al*., 2013; Zhang *et al*., 2019a). In the present study, compared with the non‐inoculation control, separate inoculation of the four PGPR strains significantly increased the 16S rRNA gene copy numbers (2.0–2.9 times higher than that of the non‐inoculation control) and species richness (by 1.3%–4.6%) of the maize rhizosphere microbial community in the field under strip tillage (Fig. 1A and B), which were consistent with previous observations on other plant species (Fu *et al*., 2017; Zhang *et al*., 2019c). Inoculation of *Bacillus amyloliquefaciens* NJN‐6 leads to increased bacterial abundance and species richness in the rhizosphere, promotion of plant growth, and inhibition of disease in banana (Fu *et al*., 2017). Inoculation of *Bacillus velezensis* NJAU‐Z9 increases the abundance/species richness of bacteria in the rhizosphere of pepper and results in higher yield (Zhang *et al*., 2019c). The positive correlation between maize biomass/production and bacterial abundance/species richness in the maize rhizosphere in the present study demonstrated that regulation of the rhizosphere microbial community might be an indirect mechanism of PGPR to promote plant growth and production under strip tillage in response to PGPR inoculation (Fig. 1C and D), which is similar to previous findings (Garbeva *et al*., 2004; Bonanomi *et al*., 2010; Hu *et al*., 2017; Chen *et al*., 2020).

In addition, PGPR inoculation in the present study obviously changed the structure of bacterial communities in the maize rhizosphere as detected using MiSeq high‐throughput sequencing technology (Figs 2 and 3; Table S6). Previous studies have reported that PGPR inoculation may regulate the composition of the host rhizobacterial communities (Zhang *et al*., 2019c, 2019b,2019c, 2019b), and different inoculants have different effects on root‐associated bacterial communities, which may indirectly affect plant growth (Gadhave *et al*., 2018; Wang *et al*., 2018). In the current study, the abundance of certain bacterial populations in the maize rhizosphere was decreased or increased to various degrees after inoculation with the four PGPR strains (Fig. 3, Tables S6 and S7). In addition, LEfSe analysis revealed that the bacteria enriched by maize rhizosphere were different among the treatments (Fig. 4, Table S8). Notably, some similarities were observed after inoculation of the test strains, such as the generally increased relative abundance of *Acidobacteria*_Subgroup_6, *Chloroflexi_*KD4_96, and *Verrucomicrobiae* at the class level and unclassified_c_*Acidobacteria*_Subgroup_6 and *Mucilaginibacter* at the genus level (Tables S6 and S7). Correlation analysis revealed that these bacteria were positively correlated with plant growth traits and grain yield of maize (Fig. 5). Tao *et al*. (2017) reported similar results in that *Acidobacteria* and *Verrucomicrobia* were significantly positively correlated with grain yield of maize after fertilization with different green manures (*P* < 0.01). Indeed, these bacteria possess certain properties that are beneficial to the soil or plants. For example, *Acidobacteria*, which is among the most abundant phyla in the plant rhizosphere, plays an important role in the soil carbon and N cycles (Lee *et al*., 2008; Jiménez *et al*., 2012). Kielak *et al*. (2016) observed that inoculation with three *Acidobacteria* strains increased the root length, root and stem biomass, and number of lateral roots and root hairs of *Arabidopsis thaliana*. *Verrucomicrobia* can be specifically enriched in the rhizosphere of maize and co‐evolved with maize for hundreds of years with a diverse metabolic capacity (Aguirre‐von‐Wobeser *et al*., 2018), which may be associated with maintenance of the homoeostasis of bacterial populations in the maize rhizosphere, and has been shown to form beneficial interactions with plant roots (Fierer *et al*., 2013; Nunes da Rocha *et al*., 2013; Aguirre‐von‐Wobeser *et al*., 2018). *Chloroflexi*_KD4_96 belongs to the *Chloroflexi* phylum, which perform a wide range of metabolic activities and ecological functions, such as fermentation, anaerobic photosynthesis, nitrite oxidation, reduction and dehalogenation (Yamada and Sekiguchi, 2009; Krzmarzick *et al*., 2012; Sorokin *et al*., 2012). Belonging to the *Bacteroidetes* phylum, *Mucilaginibacter* can produce large quantities of extracellular polysaccharides, which can protect the roots of crops. in addition, the extracellular polysaccharides can improve the rhizosphere microenvironment and promote absorption of trace elements (Danhorn and Fuqua, 2007; Madhaiyan *et al*., 2010). On account of these properties, *Mucilaginibacter* are a promising target for screening of high‐efficiency PGPR.

## Conclusions

This study showed that under conservation tillage (strip tillage) in a cold region, inoculation with PGPR may significantly promote the early growth rate of maize and ultimately increase grain yield. The PGPR strains significantly increased the abundance and species richness of bacteria in the maize rhizosphere, shifted the composition of rhizosphere bacterial communities and increased the relative abundance of beneficial bacterial populations. These changes were positively correlated with early growth traits and grain yield of maize, indicating that a favourable rhizosphere bacterial community was important for plant growth and grain production. Thus, PGPR inoculation is effective in increasing maize productivity under a conservation tillage‐planting system in a cold region.

## Experimental procedures

### Bacterial isolation from the rhizosphere and PGPR selection

For rhizobacterial isolation, maize roots together with soil were collected in June 2017 from the experimental field in Lishu County, Jilin Province, China (124.13°E, 43.34°N) where maize is a traditional crop. The sampled roots and soil were transported to the laboratory on ice. Rhizosphere soil was prepared by shaking off the loosely attached soil particles; then, the root‐adhering soil particles were brushed off and used for preparation of a decimal dilution in sterile water up to 10^−6^. Aliquots of 0.1 ml of the final three dilutions were spread separately onto Luria–Bertani (LB) agar plates and incubated at 30°C for 48 h. Single colonies with different morphology were picked up for re‐streaking on the same medium to obtain the pure culture (Somasegaran and Hoben, 1994; Majeed *et al*., 2015).

The plant growth‐promoting traits of the isolates were evaluated with corresponding methods, consisting of production of IAA (Bric *et al*., 1991; Khalid *et al*., 2004), phosphate solubilization (Vyas *et al*., 2007), production of siderophores (Schwyn and Neilands, 1987), potassium solubilization (Zhang and Kong, 2014), nitrogen fixation capacity (Dobereiner *et al*., 1976) and biocontrol capacity against *Fusarium graminearum*, *F. proliferatum*, *F. verticillioides* and *F. boothii* (Gupta *et al*., 2001). All tests were performed in triplicate by incubation at 30°C for 48–96 h. Isolates with strong plant growth‐promoting traits were selected as an inoculant to evaluate their effects on growth and production of maize grown in the same area. Four isolates that significantly improved the growth and yield of maize were further identified by construction of a neighbour‐joining phylogenetic tree using mega7.0 (Kumar *et al*., 2016), based on 16S rRNA gene sequences amplified with the universal bacterial primers 27F and 1492R (Frank *et al*., 2008). The four strains A15, A28, A55 and P24 were chosen for inoculation tests in this study.

### Experimental site and design

The field experiment was conducted in the same area of the PGPR isolation. The soil in the experimental field is a typical black soil belonging to the Udolls soil type (Soil Survey Staff, 2014). The annual average temperature and rainfall are 8.3°C and 692.2 mm respectively (data from the Meteorological Bureau of Lishu County). In the field, maize plants have been strip cultivated for four years using the following practices: (i) the previous maize crop was harvested mechanically, and the straw was crushed and mulched on the soil surface in winter; and (ii) in spring, a strip‐till machine was used to prepare the straw‐free seedling belt for sowing (Mi *et al*., 2018). The planting system comprised continuous monocropping of maize and one‐year maturation.

On 3 May 2019, seeds of maize ‘Xian Yu 335’ were sown. The basic characteristics of the soil (0–20 cm) were organic matter 15.8 g kg^−1^, total N 1.30 g kg^−1^, available P 38.2 mg kg^−1^, available K 222.4 mg kg^−1^ and pH 5.48. The experiment used a complete randomized design with five treatments in four repetitions, including four inoculation treatments and one non‐inoculation control (CK). Each plot was 5 m long and 3 m wide with a planting density of 70 000 plants ha^−1^. All treatments were fertilized with 180 kg N ha^−1^, 60 kg P_2_O_5_ ha^−1^ and 60 kg K_2_O ha^−1^. Chemical fertilizers were applied in the form of urea, superphosphate and potassium chloride; all were applied to the soil as a base fertilizer before sowing.

After sowing, dry weather was encountered and sprinkler irrigation was applied on 10 May to ensure that the soil was suitable for PGPR growth and reproduction. The daily precipitation and temperature during the maize planting period and the entire growth cycle are shown in Figure S3 (data from the Meteorological Bureau of Lishu County). The grains were harvested on 3 October 2019; no lodging occurred.

### Preparation and application of PGPR inoculants

The four PGPR strains A15, A28, A55 and P24 were cultured separately in LB broth supplemented with 80 mg l^−1^ L‐tryptophan (Libbert and Risch, 1969) to the log phase (10^10–11^ cfu ml^−1^) at 30°C with shaking of 180 r.p.m. For the inoculation treatments, maize seeds were uniformly dressed separately with the four cultures (inoculants). Seeds inoculated with sterile liquid medium were used as the non‐inoculation control. All inoculated seeds were dried in shade, then sown manually in soil.

### Plant growth and yield analysis

At the jointing stage (50 days after seedling emergence, the eighth leaf expanded), three plants were randomly sampled from each plot. Plant height and the maximum length/width of leaves were measured, and then, the shoot was excised just above the ground, dried and weighed. Plant height was the natural distance from the ground to the highest point of the leaves. Green leaf area = length × width × 0.75 (Gallais *et al*., 2006). The leaf area index (LAI) was measured as the total green leaf area per unit of soil area (Xia *et al*., 2016).

At the maturation stage, two unsampled rows were selected from each plot for grain yield measurement, and the number of ears, number of grains per ear and hundred‐grain dry weight were recorded.

### Root and rhizosphere soil sampling

At the jointing stage (50 days after seedling emergence), after the shoot sample was excised, the roots were separated from the soil by vigorous shaking and the soil particles tightly adhering to the roots (<2 mm) were collected as the rhizosphere soil. The rhizosphere soil collected from three plants in the same plot was mixed to form a compiled sample, which represented one of the four replicates of each treatment. And 4 compiled samples from a total of 12 plants were used for each treatment. All soil samples stored at −80°C. The roots were collected and washed, dried, and weighed.

### DNA extraction and quantitative PCR analysis

Rhizosphere soil DNA was extracted from 0.5 g of each replicate soil sample using the FastDNA^®^ Spin Kit for Soil (MP Biomedical, Solon, OH, USA) and purified using the PowerClean^®^ DNA Clean‐up Kit (MoBio Laboratories, Carlsbad, CA, USA) in accordance with the manufacturer’s instructions. The DNA quality was assessed by electrophoresis in 1% (w/v) agarose gel.

The V3–V4 region of the 16S rRNA gene was amplified with the primers 341F (5′‐CCTAYGGGRBGCASCAG‐3′) and 806R (5′‐GGACTACNNGGGTATCTAAT‐3′) (Yu *et al*., 2005) for quantification of the total bacteria using the ChamQ™ SYBR^®^ Color qPCR Master Mix (2×) (Vazyme Biotech, Nanjing, China). The quantitative real‐time PCR (qPCR) conditions were as follows: 95°C for 5 min; followed by 35 cycles at 95°C for 30 s, primer annealing at 56°C for 30 s, and template extension at 72°C for 40 s. Assays were conducted on a LineGene 9600 Plus Real‐Time PCR system in a 20 μl reaction volume containing 10 μl of ChamQ™ SYBR^®^ Color qPCR Master Mix (2×), 2 μL template DNA, 0.4 μL of 5 μM primer F, 0.4 μL of 5 μM primer R, and 7.2 μl nuclease‐free water. Standard curves were generated using a 10‐fold serial dilution of a plasmid containing the correct copy of the *Escherichia coli* 16S rRNA gene. All qPCR reactions were run in triplicate. The specificity of PCR products was checked by melt curve analysis, and the amplification efficiency was 92.05% (*R* ^2^ = 0.999).

### Illumina MiSeq sequencing and sequence preprocessing

The V3–V4 region of the 16S rRNA gene was amplified using the same primers as used for the qPCR assay. A barcode was added to each DNA sample to distinguish the different samples. The purified PCR products were quantified using a QuantiFluor™ Single‐Tube Fluorometer (Promega, Madison, WI, USA). Sequencing was performed on an Illumina MiSeq PE300 platform by the Majorbio Bio‐Pharm Technology, Shanghai, China. The raw reads were deposited in the NCBI Sequence Read Archive (SRA) database (accession number: SRP268158).

Raw 16S rRNA gene sequencing reads were demultiplexed and processed using qiime (v 1.8.0) (Caporaso *et al*., 2010). The remaining high‐quality sequences were clustered into OTUs at 97% similarity cut‐off using uparse software (v 7.1) (Edgar, 2013). The taxonomy of unique OTUs was annotated using the RDP classifier (http://rdp.cme.msu.edu/) against the SILVA Ref database (Release132/16s) at a confidence level of 70%. The alpha diversity of bacteria was calculated using mothur (v 1.39.5) (Schloss *et al*., 2009), and the rarefaction curves were constructed with R (v 3.6.1) software.

### Data analysis

Statistical analyses were performed using IBM SPSS Statistics for Windows 21.0. The effects of PGPR inoculation on plant growth and grain yield, and on copy numbers of the 16S rRNA gene, number of OTUs, and the predominant bacteria in the rhizosphere were determined by one‐way ANOVA followed by Duncan's multiple range test (*P* <  0.05). The linear correlation between the copy numbers of the 16S rRNA gene/number of OTUs and grain yield was estimated using the *ggplot2* package in R software (v 3.6.1) (Wickham, 2009).

The differences in bacterial community composition among all treatments were analysed by PCoA and NMDS based on Bray–Curtis distances. The statistical significance of differences in all treatments was assessed by Anosim using the *vegan* package in R software (v 3.6.1) (Dixon, 2003). Linear discriminant analysis (LDA > 2) coupled with the LEfSe technique was performed to identify significantly different biomarkers at the genus level among the treatments (Segata *et al*., 2011). In addition, Spearman correlation coefficients between the predominant bacteria and plant growth traits/grain yield were calculated.

## Conflicts of interest

None declared.

## Supporting information


**Table S1.** Plant‐growth‐promoting traits detected in the 14 isolates from the maize rhizosphere in this study.
**Table S2.** Effects of inoculation with the test plant‐growth‐promoting rhizobacteria strains on plant growth traits at the seedling, jointing, and flowering stages of maize under two fertilization areas in 2018.
**Table S3.** Effects of inoculation with the test plant‐growth‐promoting rhizobacteria strains on grain yield and yield components at the maturation stage of maize under two fertilization areas in 2018.
**Table S4.** Sample sequence information and *α*‐diversity of the bacterial communities.
**Table S5.** Significance tests of the different inoculant effects on rhizosphere bacterial community compositions by Anosim with 999 permutations.
**Table S6.** The 13 predominant bacterial classes (relative abundance > 1%) and variation in abundance of the bacteria after inoculation with the test plant‐growth‐promoting rhizobacteria strains compared with the non‐inoculation control.
**Table S7.** The 15 most abundant bacterial genera and variation in abundance of the bacteria after inoculation with the test plant‐growth‐promoting rhizobacteria strains compared with the non‐inoculation control.
**Table S8.** Significantly different biomarker bacteria at the genus level among the treatments with linear discriminant analysis (LDA) > 2.
**Fig. S1.** Neighbor‐joining phylogenetic tree based on the 16S rRNA gene sequences of the tested four plant‐growth‐promoting rhizobacteria strains and closely related reference strains. GenBank accession numbers are within brackets; The 16S rRNA gene sequences of *Halorubrum coriense* Ch2^T^ and *Halorubrum alkaliphilum* DZ‐1^T^ were used as an outgroup; Bootstrap values > 60% from 1000 replicates are indicated at branches; Bar, 0.05 substitutions per nucleotide position.
**Fig. S2.** Rarefaction curves based on the *S*_obs_ index (a) and Shannon index (b) for each treatment. *S*_obs_ indicate the observed richness; CK: non‐inoculation control; A15, A28, A55, and P24 present the inoculation treatments with strains A15, A28, A55, and P24, respectively.
**Fig. S3.** Daily precipitation and temperature during the maize growth period in 2019 in Lishu County (source: Meteorological Bureau of Lishu County).

## Data Availability

The obtained sequences were submitted to the NCBI SRA database under accession number SRP268158.
