# Supplementary material for: Effectsof growth‐promoting rhizobacteria on maize growth and rhizosphere microbial community under conservation tillage in Northeast China
Source: Microb Biotechnol. 2020 Nov 9;14(2):535–50. doi: 10.1111/1751-7915.13693 (PMC7936301; doi:10.1111/1751-7915.13693)
Supplement: Supplementary file 1 — Table S1. Plant‐growth‐promoting traits detected in the 14 isolates from the maize rhizosphere in this study. Table S2. Effects of inoculation with the test plant‐growth‐promoting rhizobacteria strains on plant growth traits at the seedling, jointing, and flowering stages of maize under two fertilization areas in 2018. Table S3. Effects of inoculation with the test plant‐growth‐promoting rhizobacteria strains on grain yield and yield components at the maturation stage of maize under two fertilization areas in 2018. Table S4. Sample sequence information and α‐diversity of the bacterial communities. Table S5. Significance tests of the different inoculant effects on rhizosphere bacterial community compositions by Anosim with 999 permutations. Table S6. The 13 predominant bacterial classes (relative abundance > 1%) and variation in abundance of the bacteria after inoculation with the test plant‐growth‐promoting rhizobacteria strains compared with the non‐inoculation control. Table S7. The 15 most abundant bacterial genera and variation in abundance of the bacteria after inoculation with the test plant‐growth‐promoting rhizobacteria strains compared with the non‐inoculation control. Table S8. Significantly different biomarker bacteria at the genus level among the treatments with linear discriminant analysis (LDA) > 2. Fig. S1. Neighbor‐joining phylogenetic tree based on the 16S rRNA gene sequences of the tested four plant‐growth‐promoting rhizobacteria strains and closely related reference strains. GenBank accession numbers are within brackets; The 16S rRNA gene sequences of Halorubrum coriense Ch2T and Halorubrum alkaliphilum DZ‐1T were used as an outgroup; Bootstrap values > 60% from 1000 replicates are indicated at branches; Bar, 0.05 substitutions per nucleotide position. Fig. S2. Rarefaction curves based on the S obs index (a) and Shannon index (b) for each treatment. S obs indicate the observed richness; CK: non‐inoculation control; A15, A28, A55, and P24 present t [file MBT2-14-535-s001.docx]

Table S1. Plant-growth-promoting traits detected in the 14 isolates from the maize rhizosphere in this study

| Isolates | Genus | Growth promoting traits | | | | | |
| --- | --- | --- | --- | --- | --- | --- | --- |
|  |  | Indole-3-acetic acid synthesis | Phosphate solubilizing | Siderophore releasing | Potassium solubilizing | Nitrogen fixation | Biocontrol |
| **A15** | *Sinorhizobium* | + | - | - | - | - | - |
| **A55** | *Sphingomonas* | + | - | + | - | - | - |
| **A28** | *Bacillus* | + | - | + | + | - | - |
| **P24** | *Enterobacter* | + | + | - | - | - | - |
| A75 | *Rhizobium* | + | - | + | - | - | - |
| P23 | *Pantoea* | + | + | - | - | - | - |
| P02 | *Rhizobium* | - | + | - | - | - | - |
| P01 | *Rhizobium* | - | + | - | - | - | - |
| N39 | *Azospirillum* | + | - | - | - | + | - |
| N79 | *Azospirillum* | + | - | - | - | + | - |
| K21 | *Rhizobium* | - | - | - | + | - | - |
| K00 | *Bacillus* | - | - | - | + | - | - |
| J37 | *Bacillus* | - | - | - | - | - | + |
| J03 | *Bacillus* | - | - | - | - | - | + |

"+" the isolate possesses the function, "-" the isolate lacks the function; Bold font indicates the strains used in this experiment.

Table S2. Effects of inoculation with the test plant-growth-promoting rhizobacteria strains on plant growth traits at the seedling, jointing, and flowering stages of maize under two fertilization areas in 2018

| Areas | Treatments | Seedling | | | Jointing | | | Flowering | | |
| --- | --- | --- | --- | --- | --- | --- | --- | --- | --- | --- |
|  |  | Plant height  (cm plant^-1^) | Shoot dry weight  (g plant^-1^) | Root dry weight  (g plant^-1^) | Plant height  (cm plant^-1^) | Shoot dry weight  (g plant^-1^) | Leaf area index | Plant height  (cm plant^-1^) | Shoot dry weight  (g plant^-1^) | Leaf area index |
| Nitrogen reduction | A15 | 46.4±0.9bc | **2.94±0.18b** | **0.37±0.01ab** | **169±1ab** | **38.8±1.8ab** | 2.94±0.09abc | **235±3ab** | **118±3a** | **5.55±0.14ab** |
|  | A55 | 47.3±0.9abc | **2.88±0.21b** | **0.35±0.02b** | **168±2ab** | 35.9±1.2bc | 2.85±0.08abc | 230±2bc | **116±3a** | 5.36±0.13bc |
|  | A28 | **49.8±1.4a** | **3.51±0.18a** | **0.42±0.02a** | **170±2ab** | **39.4±1.1ab** | **2.98±0.11ab** | **233±1ab** | **118±3a** | **5.53±0.11ab** |
|  | P24 | **49.7±1.1a** | **3.41±0.13a** | **0.40±0.02a** | **167±1b** | **36.9±1.3b** | 2.81±0.07bc | 232±2abc | **116±3a** | 5.35±0.11bc |
|  | CK1 | 44.9±1.0c | 2.19±0.08c | 0.27±0.02c | 162±1c | 32.3±1.2c | 2.68±0.10c | 223±2c | 104±2b | 5.05±0.15c |
|  | CK2 | **48.6±0.8ab** | **3.30±0.06ab** | **0.41±0.01a** | **171±1a** | **42.8±1.8a** | **3.10±0.06a** | **237±1a** | **122±3a** | **5.85±0.06a** |
| Phosphorus reduction | A15 | 44.1±2.0ab | **2.61±0.13b** | **0.33±0.02b** | **163±2b** | **34.9±1.7b** | 2.59±0.14bc | **236±1a** | **118±4a** | **5.61±0.12ab** |
|  | A55 | 44.0±1.8ab | 2.42±0.23bc | **0.34±0.02b** | **160±3b** | **34.9±1.9b** | 2.68±0.15bc | 233±2ab | **117±3a** | **5.53±0.08b** |
|  | A28 | 45.5±1.7ab | **2.64±0.20b** | **0.36±0.02b** | **161±2b** | **36.6±1.5b** | **2.86±0.14ab** | **237±2a** | **119±3a** | **5.50±0.08b** |
|  | P24 | 44.3±0.7ab | 2.48±0.12bc | **0.32±0.01b** | **158±2b** | **35.2±1.0b** | 2.57±0.07bc | **235±2a** | **115±4a** | **5.48±0.14b** |
|  | CK1 | 41.6±1.2b | 2.09±0.09c | 0.27±0.01c | 152±1c | 30.2±0.9c | 2.34±0.07c | 229±2b | 103±3b | 5.14±0.08c |
|  | CK2 | **48.6±0.8a** | **3.30±0.06a** | **0.41±0.01a** | **171±1a** | **42.8±1.8a** | **3.10±0.06a** | **237±1a** | **122±3a** | **5.85±0.06a** |

Data are the average ± standard error (*n* = 4); Nitrogen reduction: reduction of 50% nitrogen fertilizer (120 kg N ha^−1^), 85 kg P_2_O_5_ ha^−1^, and 67.5 kg K_2_O ha^−1^; Phosphorus reduction: reduction of 100% phosphorus fertilizer (0 kg P_2_O_5_ ha^−1^), 240 kg N ha^−1^, and 67.5 kg K_2_O ha^−1^; Chemical fertilizers were applied in the form of urea, superphosphate, and potassium chloride; CK1: non-inoculation control in the nitrogen reduction and phosphorus reduction areas; CK2: non-inoculation control in conventional fertilization area (240 kg N ha^−1^, 85 kg P_2_O_5_ ha^−1^, and 67.5 kg K_2_O ha^−1^); A15, A28, A55, and P24 present the inoculation treatments with strains A15, A28, A55, and P24, respectively; Different letters in the same column in a particular fertilized area indicate a significant difference among treatments (*P* = 0.05); Bold font indicates a significantly higher value than the non-inoculation control CK1 (*P* < 0.05).

Table S3. Effects of inoculation with the test plant-growth-promoting rhizobacteria strains on grain yield and yield components at the maturation stage of maize under two fertilization areas in 2018

| Treatments | Nitrogen reduction | | | | Phosphorus reduction | | | |
| --- | --- | --- | --- | --- | --- | --- | --- | --- |
|  | Ear numbers  (10^4^ ha^-1^) | Grain numbers  per ear | Hundred-grain dry weight (g) | Yield  (10^3^ kg ha^-1^) | Ear numbers  (10^4^ ha^-1^) | Grain numbers  per ear | Hundred-grain dry weight (g) | Yield  (10^3^ kg ha^-1^) |
| A15 | 6.58±0.08ab | 530±13a | **31.8±0.5a** | **12.0±0.1ab** | 7.00±0.25a | 517±16a | 31.8±0.3ab | 12.3±0.3ab |
| A55 | 6.58±0.08ab | 520±23a | **32.1±0.3a** | **12.4±0.4ab** | 6.67±0.17a | 533±20a | 31.7±0.3ab | 12.4±0.5ab |
| A28 | 6.58±0.17ab | 526±18a | **32.2±0.3a** | **12.1±0.4ab** | 6.92±0.22a | 533±13a | 31.5±0.3b | 12.2±0.5ab |
| P24 | 6.50±0.14ab | 526±15a | **32.2±0.4a** | 11.6±0.4bc | 6.83±0.08a | 551±10a | 31.8±0.2ab | **12.6±0.1a** |
| CK1 | 6.42±0.08b | 502±22a | 30.4±0.2b | 10.8±0.2c | 6.83±0.08a | 512±16a | 30.8±0.3b | 11.0±0.3b |
| CK2 | **6.83±0.08a** | 536±13a | **32.8±0.6a** | **12.8±0.2a** | 6.83±0.08a | 536±13a | **32.8±0.6a** | **12.8±0.2a** |

Data are the average ± standard error (*n* = 4); Nitrogen reduction: reduction of 50% nitrogen fertilizer (120 kg N ha^−1^), 85 kg P_2_O_5_ ha^−1^, and 67.5 kg K_2_O ha^−1^; Phosphorus reduction: reduction of 100% phosphorus fertilizer (0 kg P_2_O_5_ ha^−1^), 240 kg N ha^−1^, and 67.5 kg K_2_O ha^−1^; Chemical fertilizers were applied in the form of urea, superphosphate, and potassium chloride; CK1: non-inoculation control in the nitrogen reduction and phosphorus reduction areas; CK2: non-inoculation control in the conventional fertilization area (240 kg N ha^−1^, 85 kg P_2_O_5_ ha^−1^, and 67.5 kg K_2_O ha^−1^); A15, A28, A55, and P24 present the inoculation treatments with strains A15, A28, A55, and P24, respectively; Different letters in the same column indicate a significant difference among treatments (*P* = 0.05); Bold font indicates a significantly higher value than the non-inoculation control CK1 (*P* < 0.05).

Table S4. Sample sequence information and *α*-diversity of the bacterial communities

| Samples | Sequences | OTUs | Chao1 | Shannon | Simpson | Coverage |
| --- | --- | --- | --- | --- | --- | --- |
| CK_1 | 32565 | 1449 | 1754.07 | 5.8403 | 0.0136 | 0.9820 |
| CK_2 | 38728 | 1457 | 1747.61 | 5.9408 | 0.0095 | 0.9823 |
| CK_3 | 47687 | 1508 | 1766.03 | 5.9789 | 0.0096 | 0.9826 |
| CK_4 | 44001 | 1507 | 1864.47 | 6.0804 | 0.0074 | 0.9815 |
| A15_1 | 33242 | 1497 | 1782.60 | 6.0489 | 0.0071 | 0.9821 |
| A15_2 | 40936 | 1494 | 1762.89 | 5.9528 | 0.0087 | 0.9819 |
| A15_3 | 20997 | 1479 | 1743.82 | 6.0323 | 0.0081 | 0.9826 |
| A15_4 | 49579 | 1529 | 1769.31 | 6.1064 | 0.0065 | 0.9831 |
| A28_1 | 53812 | 1531 | 1805.74 | 6.0704 | 0.0072 | 0.9822 |
| A28_2 | 33874 | 1523 | 1776.81 | 6.0110 | 0.0091 | 0.9837 |
| A28_3 | 37429 | 1518 | 1731.66 | 5.9132 | 0.0105 | 0.9836 |
| A28_4 | 42680 | 1535 | 1753.45 | 6.0274 | 0.0083 | 0.9837 |
| A55_1 | 30000 | 1500 | 1767.72 | 5.9319 | 0.0088 | 0.9835 |
| A55_2 | 52437 | 1537 | 1765.47 | 6.0881 | 0.0077 | 0.9841 |
| A55_3 | 38466 | 1403 | 1640.37 | 5.8380 | 0.0105 | 0.9842 |
| A55_4 | 52311 | 1577 | 1772.63 | 6.2116 | 0.0063 | 0.9844 |
| P24_1 | 35671 | 1468 | 1743.79 | 5.9632 | 0.0084 | 0.9824 |
| P24_2 | 39675 | 1424 | 1695.10 | 5.8974 | 0.0099 | 0.9826 |
| P24_3 | 42961 | 1529 | 1789.63 | 5.9633 | 0.0090 | 0.9826 |
| P24_4 | 50166 | 1475 | 1769.63 | 5.8695 | 0.0107 | 0.9819 |

Table S5. Significance tests of the different inoculant effects on rhizosphere bacterial community compositions by Anosim with 999 permutations

|  | *R* | *P* |
| --- | --- | --- |
| CK vs A15 | 0.542 | **0.034** |
| CK vs A28 | 0.583 | **0.034** |
| CK vs A55 | 0.375 | 0.066 |
| CK vs P24 | 0.685 | **0.047** |
| A15 vs A28 | 0.778 | 0.098 |
| A15 vs A55 | 0.490 | **0.034** |
| A15 vs P24 | 0.740 | **0.034** |
| A28 vs A55 | 0.074 | 0.494 |
| A28 vs P24 | 0.259 | 0.296 |
| A55 vs P24 | 0.370 | 0.098 |
| Total | 0.679 | **0.001** |

An *R* value near +1 means that there is dissimilarity between the treatments, while an *R* value near 0 indicates no significant dissimilarity between the treatments. *P* Values in bold indicate significant dissimilarity (*P* < 0.05). CK: non-inoculation control; A15, A28, A55, and P24 present the inoculation treatments with strains A15, A28, A55, and P24, respectively; Total: all treatments.

Table S6. The 13 predominant bacterial classes (relative abundance > 1%) and variation in abundance of the bacteria after inoculation with the test plant-growth-promoting rhizobacteria strains compared with the non-inoculation control

| Classes | CK | A15 | | A28 | | A55 | | P24 | |
| --- | --- | --- | --- | --- | --- | --- | --- | --- | --- |
|  | Abundance | Abundance | Variation | Abundance | Variation | Abundance | Variation | Abundance | Variation |
| *Gammaproteobacteria* | 24.16±1.41ab | 20.43±0.75b | -15.43% | 24.57±0.25ab | 1.68% | 24.56±2.28ab | 1.66% | 25.94±0.64a | 7.35% |
| *Actinobacteria* | 13.44±1.29b | 14.07±0.47b | 4.73% | 21.36±2.38a | 58.95% | 17.74±0.86ab | 32.01% | 21.51±1.67a | 60.04% |
| *Alphaproteobacteria* | 20.74±1.12a | 14.95±1.49b | -27.94% | 15.21±0.66b | -26.68% | 16.47±1.72b | -20.58% | 14.96±0.47b | -27.86% |
| *Acidobacteria* | 10.75±1ab | 14.27±1.24a | 32.73% | 7.34±0.45b | -31.73% | 9.78±3.1ab | -9.10% | 7.43±1.16b | -30.94% |
| *Gemmatimonadetes* | 9.6±0.83a | 8.15±0.48ab | -15.04% | 6.38±1.28b | -33.52% | 7.55±1.19ab | -21.37% | 7.01±0.55ab | -26.92% |
| *Bacteroidia* | 6.22±0.84a | 5.79±0.42a | -6.83% | 7.06±0.58a | 13.54% | 5.66±0.41a | -9.01% | 5.34±0.58a | -14.18% |
| *Acidobacteria*_Subgroup_6 | 2.23±0.74a | 4.94±1.17a | 121.95% | 3.75±0.25a | 68.69% | 3.2±1.15a | 43.97% | 4.08±0.52a | 83.22% |
| *Deltaproteobacteria* | 3.2±0.17a | 2.62±0.14ab | -18.12% | 2.13±0.14b | -33.58% | 2.17±0.37b | -32.12% | 1.91±0.18b | -40.45% |
| *Verrucomicrobiae* | 1.35±0.22a | 3±0.85a | 122.40% | 2.44±0.56a | 80.94% | 2.19±0.97a | 62.50% | 2.07±0.39a | 53.71% |
| *Saccharimonadia* | 2.12±0.07ab | 1.79±0.36b | -15.62% | 1.76±0.26b | -17.03% | 2.89±0.47a | 35.95% | 1.22±0.15b | -42.39% |
| *Chloroflexi*_KD4-96 | 0.71±0.11b | 1.99±0.33a | 180.47% | 1.34±0.25ab | 88.94% | 1.28±0.52ab | 80.00% | 1.28±0.1ab | 80.00% |
| *Anaerolineae* | 0.81±0.17a | 1.12±0.31a | 39.54% | 1.15±0.15a | 43.06% | 0.9±0.25a | 11.59% | 1.32±0.59a | 63.56% |
| *Blastocatellia*_Subgroup_4 | 0.58±0.22a | 1.19±0.3a | 104.01% | 0.76±0.03a | 30.95% | 0.76±0.29a | 30.66% | 0.84±0.1a | 43.84% |

Data are the average ± standard error (*n* = 3); CK: non-inoculation control; A15, A28, A55, and P24 present the inoculation treatments with strains A15, A28, A55, and P24, respectively; Different letters in the same column indicate a significant difference among treatments (*P* < 0.05).

Table S7. The 15 most abundant bacterial genera and variation in abundance of the bacteria after inoculation with the test plant-growth-promoting rhizobacteria strains compared with the non-inoculation control

| Genera | CK | A15 | | A28 | | A55 | | P24 | |
| --- | --- | --- | --- | --- | --- | --- | --- | --- | --- |
|  | Abundance | Abundance | Variation | Abundance | Variation | Abundance | Variation | Abundance | Variation |
| *Massilia* | 7.21±0.48bc | 5.11±1.22c | -29.21% | 10.33±0.74a | 43.24% | 8.95±0.24ab | 24.13% | 10.46±0.85a | 45.09% |
| *Sphingomonas* | 9.00±0.96a | 6.37±0.80b | -29.25% | 5.45±0.80b | -39.40% | 6.15±0.62b | -31.65% | 5.70±0.55b | -36.67% |
| *Gemmatimonas* | 5.03±0.44a | 4.16±0.31ab | -17.32% | 3.33±0.55b | -33.89% | 3.99±0.61ab | -20.74% | 3.47±0.47ab | -31.04% |
| *Streptomyces* | 1.92±0.27c | 2.09±0.10bc | 8.67% | 5.79±1.51a | 201.04% | 4.35±0.38ab | 126.28% | 5.37±0.28a | 179.18% |
| unclassfied_c_*Acidobacteria_*Subgroup_6 | 2.19±0.74a | 4.89±1.17a | 122.97% | 3.73±0.25a | 70.27% | 3.17±1.14a | 44.79% | 4.03±0.52a | 83.65% |
| unclassfied_o*_Acidobacteriales* | 2.81±1.02b | 6.79±0.64a | 141.35% | 2.44±0.26b | -13.33% | 3.21±1.82b | 14.10% | 2.57±0.44b | -8.59% |
| unclassfied_f*_Betaproteobacteriales_*SC-I-84 | 4.25±0.57a | 2.98±0.18ab | -29.88% | 1.84±0.65b | -56.82% | 2.63±0.58ab | -38.20% | 2.79±0.36ab | -34.39% |
| *Candidatus_Solibacter* | 4.42±0.53a | 2.82±0.35b | -36.19% | 1.64±0.37b | -62.98% | 2.24±0.38b | -49.32% | 1.86±0.28b | -57.89% |
| unclassfied_o_*Gaiellales* | 2.42±0.09ab | 2.70±0.11a | 11.28% | 2.28±0.08b | -5.91% | 2.56±0.18ab | 5.43% | 2.50±0.09ab | 3.03% |
| *Ellin*6067 | 2.33±0.33ab | 2.44±0.03a | 4.94% | 1.21±0.33c | -48.17% | 1.47±0.24c | -37.01% | 1.59±0.15bc | -31.71% |
| unclassfied_f_*Gemmatimonadaceae* | 1.92±0.13a | 1.86±0.09a | -3.38% | 1.58±0.35a | -17.61% | 1.85±0.35a | -3.99% | 1.78±0.11a | -7.55% |
| unclassfied_o_*Saccharimonadales* | 1.91±0.08ab | 1.55±0.31b | -18.64% | 1.57±0.22b | -17.67% | 2.58±0.34a | 35.26% | 1.09±0.15b | -42.61% |
| *Mucilaginibacter* | 1.15±0.18a | 1.91±0.35a | 65.80% | 2.46±0.69a | 113.77% | 1.53±0.06a | 33.33% | 1.54±0.46a | 34.06% |
| *Rhodanobacter* | 1.80±0.56a | 1.47±0.30a | -18.35% | 1.26±0.37a | -29.84% | 1.25±0.16a | -30.40% | 1.63±0.28a | -9.55% |
| *Bryobacter* | 1.97±0.13a | 1.32±0.10b | -32.77% | 1.11±0.12b | -43.86% | 1.40±0.28b | -28.87% | 1.34±0.17b | -32.18% |

Data are the average ± standard error (*n* = 3); CK: non-inoculation control; A15, A28, A55, and P24 present the inoculation treatments with strains A15, A28, A55, and P24, respectively; Different letters in the same column indicate a significant difference among treatments (*P* < 0.05).

Table S8. Significantly different biomarker bacteria at the genus level among the treatments with linear discriminant analysis (LDA) > 2

| Treatments | Genera | Abundance (%) | LDA_value | *P* value |
| --- | --- | --- | --- | --- |
| CK | norank_f_*Micropepsaceae* | 1.69 | 3.632 | 0.039 |
|  | unclassified_f*_Gemmatimonadaceae* | 0.74 | 3.412 | 0.015 |
|  | norank_f*_Betaproteobacteriales*_A21b | 0.42 | 3.231 | 0.026 |
|  | norank_o*_Elsterales* | 0.61 | 3.194 | 0.037 |
|  | norank_f*_Acetobacteraceae* | 0.44 | 3.123 | 0.028 |
|  | *Roseiarcus* | 0.03 | 2.923 | 0.024 |
|  | norank_c*_Anaerolineae* | 0.03 | 2.885 | 0.047 |
|  | unclassified_f*_Polyangiaceae* | 0.08 | 2.722 | 0.023 |
|  | norank_o*_Deltaproteobacteria*_RCP2_54 | 0.09 | 2.702 | 0.013 |
|  | *Nitrosospira* | 0.05 | 2.657 | 0.048 |
| A15 | unclassified_f*_Methylophilaceae* | 0.04 | 2.814 | 0.037 |
|  | *Anaeromyxobacter* | 0.08 | 2.705 | 0.030 |
|  | norank_f_*Rickettsiales*_SM2D12 | 0.05 | 2.479 | 0.039 |
| A28 | *Streptomyces* | 5.79 | 4.249 | 0.029 |
|  | unclassified_f*_Enterobacteriaceae* | 0.64 | 3.465 | 0.045 |
|  | *Mesorhizobium* | 0.64 | 3.224 | 0.038 |
|  | *Pseudoduganella* | 0.22 | 2.952 | 0.032 |
|  | norank_f_*Solirubrobacterales*_67_14 | 0.30 | 2.894 | 0.028 |
|  | *Variovorax* | 0.19 | 2.762 | 0.041 |
|  | *Geodermatophilus* | 0.03 | 2.683 | 0.017 |
|  | *Achromobacter* | 0.02 | 2.654 | 0.018 |
|  | norank_c_*Chloroflexi_*Gitt_GS_136 | 0.06 | 2.634 | 0.033 |
|  | *unclassified_o_Microtrichales* | 0.05 | 2.517 | 0.047 |
| A55 | *Chujaibacter* | 2.34 | 3.973 | 0.029 |
|  | *Pseudolabrys* | 0.76 | 3.152 | 0.042 |
|  | *Devosia* | 0.59 | 3.129 | 0.033 |
|  | unclassified_f_*Micromonosporaceae* | 0.33 | 2.933 | 0.029 |
|  | *Fimbriimonas* | 0.02 | 2.705 | 0.047 |
|  | *Acidiphilium* | 0.03 | 2.635 | 0.027 |
|  | *Shimazuella* | 0.03 | 2.469 | 0.021 |
| P24 | *Massilia* | 10.46 | 4.435 | 0.024 |
|  | *Aeromicrobium* | 0.54 | 3.190 | 0.042 |
|  | *Kribbella* | 0.42 | 3.109 | 0.035 |
|  | *Duganella* | 0.31 | 2.962 | 0.042 |
|  | norank_c_*Latescibacteria* | 0.23 | 2.947 | 0.029 |
|  | norank_c_*Chloroflexi*_AD3 | 0.18 | 2.866 | 0.024 |
|  | *Actinoallomurus* | 0.12 | 2.810 | 0.028 |
|  | *Spirosoma* | 0.02 | 2.765 | 0.010 |
|  | norank_f*_Solirubrobacteraceae* | 0.07 | 2.526 | 0.013 |

CK: non-inoculation control; A15, A28, A55, and P24 present the inoculation treatments with strains A15, A28, A55, and P24, respectively.

**Fig. S1.** Neighbor-joining phylogenetic tree based on the 16S rRNA gene sequences of the tested four plant-growth-promoting rhizobacteria strains and closely related reference strains. GenBank accession numbers are within brackets; The 16S rRNA gene sequences of *Halorubrum coriense* Ch2^T^ and *Halorubrum alkaliphilum* DZ-1^T^ were used as an outgroup; Bootstrap values > 60% from 1,000 replicates are indicated at branches; Bar, 0.05 substitutions per nucleotide position.

*Sphingomonas yabuuchiae* GTC 868^T^ (AB071955)

**A55 (MN905523)**

*Sphingomonas pseudosanguinis* G1-2^T^ (AM412238)

*Sphingomonas parapaucimobilis* NBRC 15100^T^ (BBPI01000114)

*Sinorhizobium chiapanecum* ITTG S70^T^ (EU286550)

*Sinorhizobium fredii* NBRC 14780^T^ (BJNI01000207)

*Sinorhizobium americanus* CFNEI 156^T^ (LNQC01000019)

**A15 (MT956581)**

*Enterobacter cloacae* ATCC 13047^T^ (CP001918)

*Enterobacter asburiae* JCM 6051^T^ (BBED01000197)

**P24 (MN905526)**

*Enterobacter hormaechei* ATCC 49162^T^ (AFHR01000079)

*Bacillus benzoevorans* DSM 5391^T^ (D78311)

*Bacillus acidiceler* CBD 119^T^ (DQ374637)

*Bacillus megaterium* NBRC 15308^T^ (JJMH01000057)

**A28 (MN905525)**

*Halorubrum coriense* Ch2^T^ (L00922)

*Halorubrum alkaliphilum* DZ-1^T^ (AY510708)

100

100

77

100

93

60

100

99

100

100

99

100

100

0.050

**Fig. S2.** Rarefaction curves based on the *S*_obs_ index (a) and Shannon index (b) for each treatment. *S*_obs_ indicate the observed richness; CK: non-inoculation control; A15, A28, A55, and P24 present the inoculation treatments with strains A15, A28, A55, and P24, respectively.

**Fig. S3.** Daily precipitation and temperature during the maize growth period in 2019 in Lishu County (source: Meteorological Bureau of Lishu County).
